# Morphology and Olfactory Recognition of Leg Sensilla in Honeybee Workers of *Apis cerana cerana*

**DOI:** 10.3390/insects16090961

**Published:** 2025-09-12

**Authors:** Huiman Zhang, Lele Sun, Peng Wang, Jiaoxin Xie, Yuan Guo

**Affiliations:** 1 College of Animal Science, Shanxi Agricultural University, Jinzhong 030801, China; m19915323437@163.com (H.Z.); 202430647@stu.sxau.edu.cn (L.S.); w18534271561@163.com (P.W.); 2 Shanxi Key Laboratory of Animal Genetics Resource Utilization and Breeding, Jinzhong 030801, China; 3 College of Horticulture, Shanxi Agricultural University, Taiyuan 030031, China

**Keywords:** *Apis cerana cerana*, leg, morphology, sensillum, electrolegogram test, Y-tube olfactometer

## Abstract

*Apis cerana cerana* is a key social insect, and its ability to recognize chemical signals is crucial for maintaining colony homeostasis and coordinating collective behaviors, such as foraging, nursing, and defense. The legs of insects play a significant role in gustatory perception and proximity olfactory perception. In this study, the leg sensilla of *A. c. cerana* were observed by scanning electron microscopy (SEM). Two types of sensilla were observed, including sensilla trichodea (Str I, Str II, Str III, Str IV, Str V, and Str VI) and sensilla chaetica (Sch I, Sch II, and Sch III). The two unique structures of the tibial spur (Tsp I, Tsp II) and antennal brush (Abr) are carefully observed. The electrophysiological responses of workers at different ages to diverse chemical compounds were measured via electrolegogram (ELG) recordings on their legs. The results showed that 1-day-old *A. c. cerana* was more sensitive to nonanal; 10-day-old and 25-day-old *A. c. cerana* were more sensitive to ocimene. The results of behavioral responses showed that nonanal and ocimene can significantly attract 10-day-old workers of *A. c. cerana*. This study establishes a foundation for further exploration of the mechanisms by which the legs of *A. c. cerana* facilitate colony-level communication through chemical signals. It also provides an important theoretical basis for understanding their social organization and information transmission.

## Simple Summary

As a native species in China; the survival and reproduction of *Apis cerana cerana* are heavily dependent on chemical signals. In the process of chemical communication, the antennae of bees serve as the primary sensing organs, and the legs also play an important role in the reception and transmission of chemical signals. In our study, scanning electron microscopy (SEM) was used to observe its legs, revealing two types of sensilla, and the ultrastructure of the tibial spur and the antennal brush was observed. The electrophysiological responses of the legs (ELG), along with Y-tube behavioral assays, were employed to validate the involvement of the bee’s legs in olfactory perception. The legs of bees at different ages showed varying degrees of significant response to nonanal and ocimene. Notably, 10-day-old bees exhibited a clear preference for nonanal and ocimene. These findings contribute to a deeper understanding of the mechanistic role of the honeybee leg in chemical signal detection, elucidating their functional characteristics in chemical communication, and providing experimental evidence for the study of bee behavioral responses and environmental adaptation.

## 1. Introduction

Chemical communication is crucial for the survival and reproduction activities of most insects, among which olfaction plays an important role [1,2,3]. In complex and dynamic environments, insects can detect trace pheromones through olfaction, enabling them to regulate their activities, reproduce offspring, and avoid danger on time [4,5]. These activities are mainly achieved by relying on their structurally complex and functionally sensitive sensory organs [6,7]. As the main chemical sensing organ, the antennae play a vital role in the sensing process. However, in addition to antennae, other insect organs are also involved in the process of chemical communication, including mouthparts, legs, wings, and ovipositors [8,9,10,11,12].

The legs of insects are of great significance in their perception and response to chemical signals [13]. The tarsus of the foreleg, for example, plays an important role in the oviposition preference of *Plutella xylostella* [14]. The tarsi of *Helicoverpa armigera* and *Helicoverpa assulta* also affect their feeding and oviposition behavior [15,16]. The morphology, type, and distribution characteristics of insect leg sensilla can be used as an important morphological basis for species classification [17]. In general, the legs of insects are not only their locomotive tools, but also their indispensable sensing organs in complex chemical communication.

Honeybees are one of the most important pollinators in the world and have been shown to play a key role in the pollination process of a variety of major crops. Their pollination activities cover more than 90% of crop varieties [18,19]. Age in days is an important biological factor affecting the labor division mode of honeybees [20]. Workers on their first day after emergence have not yet engaged in colony-related labor. Around 10 days of age, they primarily perform tasks such as nest cleaning and larval feeding. By the time they reach approximately 25 days of age, they transition to external tasks such as foraging [21]. *Apis cerana cerana* is a native bee population in China, which is widely distributed throughout the country. Different subspecies of *A. c. cerana* exhibit distinct ecological adaptations, reflecting their specialized capacity to thrive in varied environmental conditions [22,23,24,25,26]. A study investigated the tarsal taste of *Apis mellifera* through a combination of behavioral and electrophysiological analyses. For the first time, they comprehensively described the response of the tarsals of *A. mellifera* to sweet, salty, and bitter substances at the behavioral and electrophysiological levels [27]. A comprehensive morphological analysis of the hind leg bristles of the *A. c. cerana* has been conducted with the aim of elucidating the potential relationship between bristle morphology and pollination function [28]. In the legs of the *A. c. cerana*, proteins involved in chemical communication show different expression characteristics at different age stages, suggesting that the leg may play an important sensory and regulatory role in the chemical communication process of bees [29].

Chemical communication plays a vital role in various processes of the honeybee, including division of labor, food localization, and social immunity [30,31,32]. Isoamyl acetate, a volatile compound primarily associated with fruit aromas and identified as a component of alarm pheromone [33], has been shown to elicit positive behavioral responses in insects [34]. As a larval hunger pheromone, ocimene is perceived by workers, attracting nurse bees and increasing their larval acceptance rate, thereby eliciting a suite of brood-rearing behaviors [35,36]. The brood ester pheromone (BEP) of *A. c. cerana* larvae contains ten kinds of ester pheromones, including methyl palmitate (MP), methyl oleate (MO), methyl stearate (MS), methyl linoleate (ML), methyl linolenate (MLN), ethyl palmitate (EP), ethyl oleate (EO), ethyl stearate (ES), ethyl linoleate (EL), and ethyl linolenate (ELN) [37,38]. The BEP component can induce workers to seal larval cells and significantly promote the behavior of workers feeding queen larvae [39]. Plant-emitted volatile organic compounds (VOCs) are widely established as critical ecological mediators shaping pollinator foraging behavior [40], among which ethyl hexanoate, α-farnesene, linalool, and nonanal represent frequently encountered volatile semiochemicals in flowering plants [41,42,43].

The recognition of semiochemicals in honeybees is primarily mediated by various chemosensory organs. While extensive research has focused on antennae, studies on legs remain relatively limited, constraining our understanding of the role legs play in chemical communication. This study initially employed scanning electron microscopy to observe and characterize the types of sensilla on the legs of *A. c. cerana* workers. Subsequently, electrophysiological responses to several bee pheromones and common plant volatiles of the foreleg, middle leg, and hind leg were examined. Finally, behavioral responses to compounds that elicited electrophysiological reactions were assessed using a Y-tube olfactometer. Integrating morphological, physiological, and behavioral evidence, this study offers critical insights into the role of honeybee legs in olfactory recognition and establishes a foundational framework for future investigations into legs chemoreception in honeybees.

## 2. Materials and Methods

### 2.1. Insects

*A. c. cerana* were raised in the experimental beekeeping farm of the Animal Science and Technology Experiment Station of Shanxi Agricultural University. One brood comb was selected from three healthy bee colonies and placed in an incubator (75–80% RH; 34 ± 0.4 °C). Mark newly emerged workers’ dorsal thorax with non-toxic paint and place them back in the cell until sampling. The 1-day-old workers were marked and put into the same beehive. All three pairs of legs of workers were collected at 1, 10, and 25 d, and a total of 330 bees were used in the experiment.

### 2.2. Scanning Electron Microscope (SEM) Observations of Legs

The legs were carefully excised from the thoraxes of the six forager bees and placed in a 1.5 mL centrifuge tube, categorized according to the fore, middle, and hind legs. Subsequently, they underwent a graded dehydration process using 70%, 80%, 90%, 95%, and 100% anhydrous ethanol. Then complete drying was performed using a critical point dryer (LEICA EM CPD300, Leica-microsystem; Shanghai, China). Samples were prepared and placed in a fully automatic ion sputtering instrument (JEC-3000FC, JEOL, Tokyo, Japan). Finally, the legs were observed using a scanning electron microscope (JSM-IT300, JEOL, Tokyo, Japan).

### 2.3. Electrophysiological Reactions to Testing Compounds

**Figure 1:**
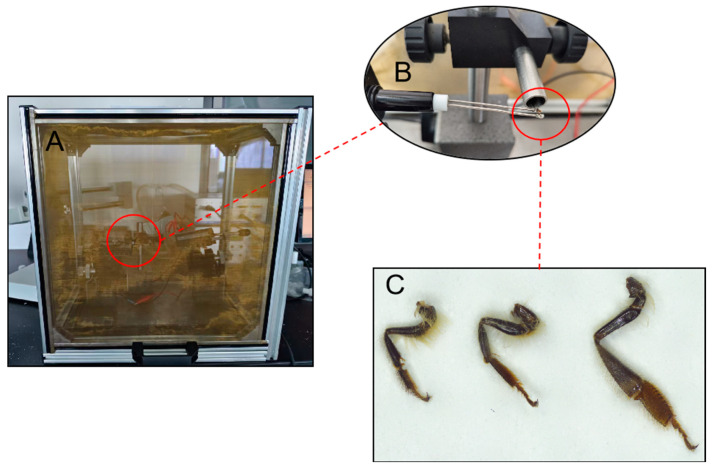
The schema of ELG. (**A**) ELG instruments. (**B**) The leg was connected to the potential probe. (**C**) Whole legs (from left to right in turn: foreleg, middle leg, and hind leg).

The responses of workers’ legs to testing compounds at 1, 10, and 25 days of age were tested through physiological electrical changes in the leg (ELG). The testing compounds are derived from the components of bee pheromones and common plant volatiles (Table 1). All the testing compounds were dissolved in paraffin liquid to prepare a reagent with a concentration of 400 μg/μL. An amount of 10 μL of the reagent is taken each time and applied to filter paper strips (0.5 cm × 3 cm). The blank control consists of the same volume of paraffin. To observe electrophysiological response of the entire leg to the testing compounds, the leg was cut off from the root and connected to the potential probe with a coupling agent (Figure 1). The ELG signal was amplified by a DC/AC amplifier, recorded using Syntech IDAC2 (Syntech, Kirchzarten, Germany), and analyzed using EagPro V.2 software (Syntech, Kirchzarten, Germany). Six replicates were tested for the foreleg, middle leg, and hind leg at each age. The response of workers to volatile compounds is expressed as ELG relative response values (mean ± standard error). The calculation method for the ELG relative response value (rELG) is as follows [44]:

rELG (%) = (ELG (X) − ELG (std))/ELG (std)

In the above formula, ELG (X) is the amplitude (mV) of the ELG response to the compound, and ELG (std) is the amplitude (mV) of the ELG response to the reference liquid paraffin in each recording process.

### 2.4. Preference Selection for Testing Compounds

The Y-tube behavioral experiments were conducted in a darkroom. A red light was placed in front of the Y-shaped olfactory apparatus (each of the three arms was 18 cm long, the angle between the two arms was 70°, and the inner diameter of the tube was 2.6 cm), and the light intensity of the left and right arms was kept consistent. The outlet of the atmospheric sampling instrument (QC-1S, Beijing Municipal Institute of Labour Protection, Beijing, China) was used as the air flow source, and the air flow velocity of both arms of the olfactory instrument was set at 350 mL/min. Ventilate for 5 min in advance to ensure that there are no other impurity gases in the Y-shaped tube. An amount of 10 μL of four volatile substances (tested at a concentration of 400 μg/μL in ELG) was, respectively, dropped onto filter paper strips (0.5 cm × 3 cm) and placed in one side of the flavor source bottle. Filter paper strips containing the same volume of paraffin were placed on the other side of the flavor source bottle as controls. Place a lively *A. c. cerana* with antennae sealed with non-toxic paint at the entrance of the main arm through a Y-tube. Start timing when it has crawled over one-third of the main arm. Those that enter the side arm of the flavor source within 5 min and stay for more than 30 s are regarded as the selective treatment, and those that enter the control side arm and stay for more than 30 s are regarded as the selective control. If the test insect remains in the main arm for more than 5 min and does not enter the test arms on both sides, it is regarded as not having made a selection. For each volatile substance, 30 specimens were tested in one group. Each compound was tested in three groups, and each honeybee was used only once. To avoid interference, for every 5 insects tested, the direction of the Y-shaped tube is changed left and right once. After testing 5 heads, replace the filter paper strips and Y-shaped tubes once. To ensure the elimination of any potential interference, thoroughly rinse the Y-shaped tube with ethanol and distilled water, followed by drying. The testing compounds with obvious reactions in the ELG were selected as the test odors for the Y-tube behavior. Isoamyl acetate was set as a control to verify the reliability of the experiment. The results of subsequent behavioral experiments showed that the compound also did not cause significant behavioral responses, which confirmed the EAG results. In the Y-tube behavior experiment, only the selected *A. c. cerana* were statistically analyzed, and the selection rate of the *A. c. cerana* to the paraffin control group and the candidate odorant test group in the Y-tube behavior experiment was calculated according to the following formula:

Paraffin liquid (%): the total number of bees in the paraffin liquid/the total number of bees in the test group and the paraffin liquid × 100%.

Test compound (%): the total number of bees in the test group/the total number of bees in the test group and the paraffin liquid × 100%.

### 2.5. Statistical Analysis

The images of the morphology and SEM were processed and measured using ImageJ V.1.8 (National Institutes of Health, Bethesda, MD, USA). The naming and classification of the types of sensors on the leg’s surface were mainly described following the standards of Schneider [45], Lu [46], Zhao et al. [47], and Liu et al. [48]. During the sensor measurement, seven sensors of the same type were randomly selected and their average values were taken. The sensor length was the average value ± the standard error. One-way ANOVA was used to analyze the ELG response data of workers to testing compounds at different ages, and the least significant difference (LSD) post hoc test was performed using IBM SPSS Statistics 27.0 software (SPSS Inc., Chicago, IL, United States), with *p* < 0.05. The Y-tube behavior experiment was analyzed by chi-square test.

## 3. Results

### 3.1. SEM of the Legs

The worker possesses three distinct pairs of legs: the foreleg, middle leg, and hind leg. The legs of worker are composed of coxa, trochanter, femur, tibia, and tarsus. The foreleg length was 6.57 ± 0.52 mm, the middle leg length was 7.75 ± 0.41 mm, and the hind leg length was 10.60 ± 1.21 mm. The pollen basket is a unique structure of the hind leg, and the junctions of the tibia and pretarsus of the foreleg and middle leg also exhibit dissimilarity compared to the hind leg.

**Figure 2:**
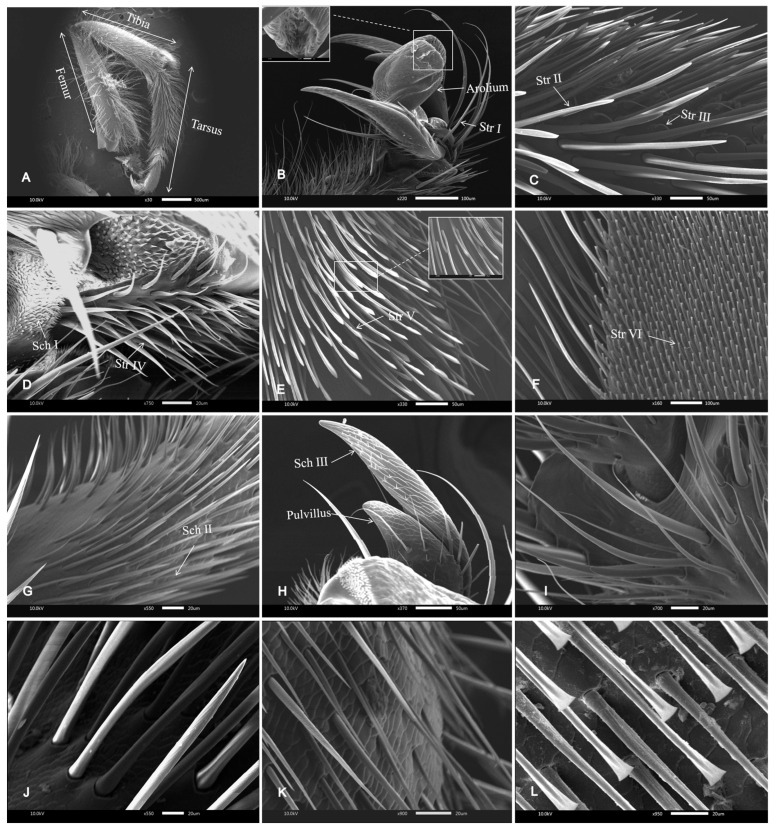
SEM of foreleg, claw, and sensilla of workers of *A. c. cerana*. (**A**) Foreleg structure. (**B**) Claw and arolium and sensilla trichodea I (Str I). (**C**) The sensilla trichodea III (Str III) is surrounded by clusters of adjacent sensilla trichodea II (Str II). (**D**) Sensilla trichodea IV (Str IV) and sensilla chaetica I (Sch I) on claws. (**E**) The sensilla trichodea V (Str V) of the foreleg. (**F**) The sensilla trichodea VI (Str VI) of the hind leg. (**G**) Sensilla chaetica II (Sch II). (**H**) Sensilla chaetica III (Sch III) on claws. (**I**) Sensilla trichodea I. (**J**) Sensilla trichodea II. (**K**) Sensilla chaetica II. (**L**) Sensilla trichodea VI.

The sensilla were mainly distributed in the femurs, tibiae, and tarsi (Figure 2A). A total of two types of receptors were observed on the worker’s legs, including sensilla trichodea and sensilla chaetica. Tibial spur and antennal brushes are the specialized structures of bee legs, and their morphological characteristics were emphatically observed.

The sensilla trichodea (Str) is the most common sensory structure of the leg. The base of the sensilla trichodea is robust, gradually tapering toward the distal end. It exhibits a distinct curvature and is characterized by spiral ridges, which are distributed across multiple regions. The Str I was 312.43 ± 14.22 μm (Figure 2B,J). The Str II was distributed in many places on the leg, and the tip was thicker than that of Str I, with a length of 212.17 ± 8.06 μm (Figure 2C,K). The Str III was comparatively low and exhibited a pronounced curvature. It was often distributed between the Str II, with a length of 46.76 ± 4.44 μm (Figure 2C). Str IV was distributed on the arolium, and the tip was thin; it had an average length of 51.76 ± 6.18 μm (Figure 2D). The Str V was only distributed to the tip of the tibia and around the pretarsus of the foreleg. The end was like a tuft, and the middle bulge was flat on both sides. There were clear markings on the trunk, with a length of 141.59 ± 4.89 μm (Figure 2E). The Str VI was only distributed on the posterior tibial segment of the hind leg. The main body of the structure was linear, exhibiting a distinct and well-defined carved pattern, while the distal end was flattened and fan-shaped, with a measured length of 89.10 ± 0.71 μm (Figure 2F,L). In addition to Str II, stripes were observed on the surface of all other types of sensilla trichodea. The base of the sensilla trichodea was located in a small epidermal fossa.

The sensilla chaetica (Sch) were straight, not curved, and the tips were thin. Based on their distinct morphological characteristics, they were classified into Sch I, Sch II, and Sch III. The Sch I was only distributed at the junction of the hind tarsi and the claw, which was dense and about 15.98 ± 0.67 μm in length (Figure 2D). Sch II was mainly distributed in the hind tarsi, close to the surface of each tarsus, and it was straight and pointed and about 82.27 ± 4.20 μm in length (Figure 2D,K); Sch III was short, common in the claws and tarsi, and was about 6.92 ± 0.37 μm long (Figure 2D). Stripes were observed exclusively on Sch II.

**Figure 3:**
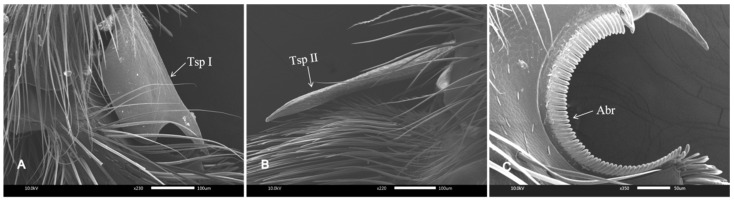
Tibial spur and groove of pretarsus. (**A**) Foreleg tibial spur, Tsp I. (**B**) Middle leg tibial spur, Tsp II. (**C**) Antennal brush, Abr, located in the groove of the pretarsus.

The tibial spur (Tsp) is a special structure that is distributed along the foreleg and middle leg of the distal tibia, both of which can move, and the hind leg has no Tsp. Tsp I of the foreleg was shaped like a knife, with a smooth surface and a diameter of 376.39 ± 8.77 μm (Figure 3A). The Tsp II of the middle leg was a long and thin spine at the thick end of the base. It was covered with a scaly texture along its length and its diameter was 491.29 ± 4.14 μm (Figure 3B). There was a half-moon-shaped groove at the base of the tarsus of the foreleg. Inside the groove, there was a row of antennal brushes (Abrs) with a thick base and a blunt end (Figure 3C). The Tsp I and the groove of the base tarsus together constitute the antenna cleaner.

### 3.2. ELG Responses of A. c. cerana

**Figure 4:**
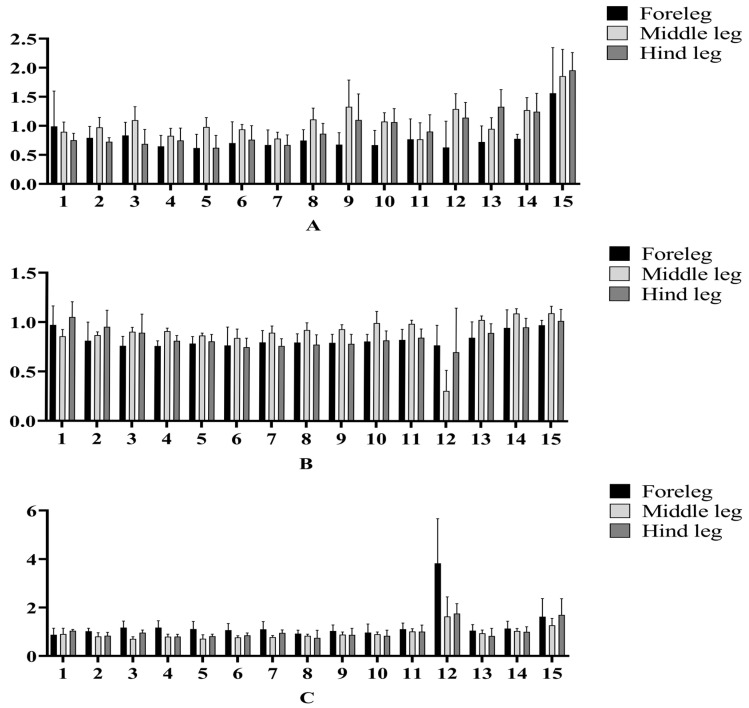
ELG responses in the leg of *A. c. cerana* at different ages. The *X*-axis labels correspond to the compound numbers in Table 1, while the *Y*-axis represents the rELG. (**A**) rELG of 1-day-old *A. c. cerana*. (**B**) rELG of 10-day-old *A. c. cerana*. (**C**) rELG of 25-day-old *A. c. cerana*.

The response of the foreleg, middle leg, and hind leg of *A. c. cerana* of different ages to 15 compounds relative to ELG is shown in Figure 4.

The results show that the legs of *A. c. cerana* at different ages can produce electrophysiological reactions to these fifteen compounds. For 1-day-old workers, the legs exhibited a pronounced response to nonanal, with the rELG value of the hind leg reaching its peak at 1.56 ± 0.39 (Figure 4A, Table 2). The rELG value of ocimene in the 10-day-old workers were largely different from that of other odorants. The rELG value of ocimene in the foreleg was 0.77 ± 0.10, the rELG value of ocimene in the middle leg was 0.31 ± 0.10, and the rELG value of ocimene in the hind leg was 0.70 ± 0.22 (Figure 4B, Table 2). In the 25-day-old group, the rELG values of ocimene and nonanal were higher than those of other odorants, and the rELG value of ocimene was also higher than that of nonanal (Figure 4C, Table 2).

According to the classification of the foreleg, middle leg, and hind leg, the rELG values of 10-day-old *A. c. cerana* legs for most candidate compounds were higher than those of their 1-day-old and 25-day-old countereparts. The change in ocimene was inconsistent with other compounds, and the rELG value was the lowest at 10 days of age, which was 0.31 ± 0.10 (Table 2).

### 3.3. Y-Tube Behavior of A. c. cerana

**Figure 5:**
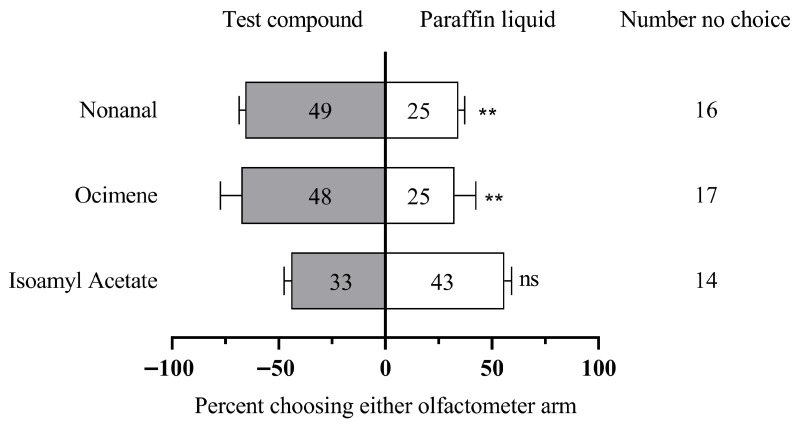
Reaction of 10-day-old *A. c. cerana* to 4 compounds in Y-tube behavioral experiment. The number represents the total number of test insects selected for the reagent. The asterisk indicates a significant difference (** *p* < 0.01, “ns” indicates no significant difference, chi-square test).

Finally, isoamyl acetate, ocimene, and nonanal were selected to conduct behavioral experiments on 10-day-old workers. We found that the total number of nonanal (χ^2^ = 9.3, df = 1, *p* = 0.002) was significantly higher than that of the control group, and the total number of ocimene (χ^2^ = 9.88, df = 1, *p* = 0.002) was significantly higher than that of the control group. There was no significant relationship between the total number of isoamyl acetate (χ^2^ = 1.14, df = 1, *p* > 0.05) and the control group (Figure 5).

## 4. Discussion

There are differences in the length and morphology of the foreleg, middle leg, and hind leg of *A. c. cerana*, which are closely related to their special roles in acquisition, tactile perception, and other physiological functions. The foreleg not only plays a crucial role in pollen collection but also functions as a tactile sensory organ, assisting bees in discerning various floral characteristics and chemical components [49]. The middle leg is slightly longer than the foreleg and features a tibial spur. In addition, sensory organs located on the middle leg are involved in the detection of chemical signals, which is crucial for the chemical communication related to foraging, navigation, and social interactions among bees [50]. The bee’s hind leg is the most distinctive among these, exhibiting greater size compared to the foreleg and middle leg, along with specialized morphological adaptations. The most typical feature is the pollen basket, which is a specialized tibia, specifically adapted for collecting and transporting pollen. In addition to fulfilling the primary function of pollen collection, the hind leg contributes significantly to the foraging efficiency of the bee through this specialized structure [51,52,53].

In the present study, a total of two types of sensilla were distributed on the leg of *A. c. cerana*, including six subtypes of sensilla trichodea and three subtypes of sensilla chaetica. These sensilla are mainly present on the tarsi. The observational findings revealed that the majority of the sensilla were sensilla trichodea. Sensilla with wall pores or pores were found in the leg sensilla of various insects [48,54,55]. However, only linear stripes were found on the surface of the sensilla in the legs of *A. c. cerana*. Ultrastructural features similar to stripes were also observed in the leg sensilla of other species [56]. In morphological studies, it is currently believed that sensilla trichodea and sensilla chaetica are mainly responsible for mechanical perception [57]. Sensilla trichodea may also help insects perceive host volatiles to find suitable oviposition sites [58]. In comparison to the antennal sensilla of *A. c. cerana* [47], the shape of these two sensilla on the antennae and legs is different, and the leg sensilla are composed exclusively of sensilla trichodea and sensilla chaetica. Most of the existing research focuses on the morphology and function of insect antennae sensilla, while systematic studies on leg sensilla are still relatively scarce [59]. To address this research gap, future studies could employ an integrated approach combining transmission electron microscopy [60] with single sensillum recording [61] to investigate the functional complementarity and coevolution of antennal and leg sensilla in honeybees.

In the ELG experiment, the rELG responses of *A. c. cerana* to test compounds at different ages were significantly different. With the increase in age, the reaction intensity of *A. c. cerana* to ocimene also increased. This indicates that age might also be one of the influencing factors of bees’ chemical communication. The rELG of ocimene shows that ocimene could cause potential physiological changes in the legs of *A. c. cerana*, and was significantly different from the control group. In this rELG, *A. c. cerana* of all ages did not respond to BEP. It may be that a single component needs to be mixed in a certain proportion to cause a reaction [62,63]. One-day-old *A. c. cerana* only showed a strong reaction to nonanal, and had no obvious reaction to the other volatiles, which needs to be further explored. The rELG of *A. c. cerana* to nonanal was higher, which was consistent with Liu’s research [64]. In his experiment, it was found that *A. mellifera* also have a very strong rEAG reaction to nonanal. In addition, nonanal can also induce strong EAG responses in other insects [65,66]. *A. c. cerana* have different sensitivities to various odor substances [67], so they have different rELG responses to substances of the same concentration.

The Y-tube behavior experiment of three odor substances was carried out on 10-day-old *A. c. cerana*, and it was found that it had obvious selection preference for nonanal and ocimene. The selection rate for isoamyl acetate did not exhibit a significant preference. According to the results of this Y-tube behavior experiment and previous studies [44], it can be seen that nonanal can cause the attraction of *A. c. cerana* and *A. mellifera*. It can also have an attractive effect on nonanal in both male and female adults of *Callosobruchus chinensis* [68]. A conceivable explanation is that nonanal, being a ubiquitous plant volatile, functions as an ecologically relevant host habitat signal capable of eliciting attractant responses across a range of herbivorous insect species. Ocimene, as an important larval pheromone, is involved in regulating the behavior of workers [69,70]. When the workers recognize it, it promotes the workers to accelerate the feeding activities of the larvae. Nurse bees demonstrate significant positive behavioral responses to elevated concentrations of ocimene via leg perception. It is postulated that the legs may serve a complementary sensory role to the antennae in detecting this compound within the light-restricted hive environment.

This study conducted a preliminary morphological investigation of the chemosensory sensilla on the legs of *A. c. cerana* workers, identifying the types and structures of leg sensilla and discussing their potential functions. Electrophysiological and behavioral assays further revealed distinct responses of the legs to specific compounds. Building on previous work that identified chemosensory genes in the legs of *A. c. cerana* [29], we propose that the leg may play a role in olfactory recognition. Nevertheless, the functional characterization of leg-specific sensilla and the associated chemosensory genes remains to be fully elucidated, necessitating further investigation to clarify their synergistic interactions with other chemosensory organs in mediating chemical communication in honeybees.

## Figures and Tables

**Table 1:** Purity and source of chemicals used in the experiments.

NO.	Chemicals	Purity%	Compound Source
1	Isoamyl Acetate	98%	Tokyo Chemical Industry Co., Ltd., Tokyo, Japan
2	Methyl Palmitate	97%	Tokyo Chemical Industry Co., Ltd., Tokyo, Japan
3	Methyl Oleate	99%	Tokyo Chemical Industry Co., Ltd., Tokyo, Japan
4	Methyl Linoleate	95%	Tokyo Chemical Industry Co., Ltd., Tokyo, Japan
5	Methyl Linolenate	98%	Tokyo Chemical Industry Co., Ltd., Tokyo, Japan
6	Methyl Stearate	95%	Tokyo Chemical Industry Co., Ltd., Tokyo, Japan
7	Ethyl Palmitate	99%	Macklin Biochemical Technology, Shanghai, China
8	Ethyl Oleate	95%	Tokyo Chemical Industry Co., Ltd., Tokyo, Japan
9	Ethyl Linoleate	97%	Tokyo Chemical Industry Co., Ltd., Tokyo, Japan
10	Ethyl Stearate	99%	Aladdin Biochemical Technology, Shanghai, China
11	Linalool	98%	Aladdin Biochemical Technology, Shanghai, China
12	Ocimene	90%	Aladdin Biochemical Technology, Shanghai, China
13	Ethyl Hexanoate	99%	Tokyo Chemical Industry Co., Ltd., Tokyo, Japan
14	Farnesene (mixture of isomers)	98%	Aladdin Biochemical Technology, Shanghai, China
15	Nonanal	96%	Aladdin Biochemical Technology, Shanghai, China

**Table 2:** Relative ELG Response Values (rELG).

Chemicals	1 Day Old			10 Days Old			25 Days Old		
	Forelegs	Middle Legs	Hind Legs	Forelegs	Middle Legs	Hind Legs	Forelegs	Middle Legs	Hind Legs
Isoamyl Acetate	0.99 ± 0.30 **A**b	0.90 ± 0.08 **A**cd	0.75 ± 0.06 **A**defg	0.97 ± 0.10 **A**a	0.86 ± 0.03 **A**de	1.05 ± 0.08 **A**a	0.88 ± 0.14 **A**b	0.91 ± 0.12 **A**bc	1.04 ± 0.03 **A**b
Methyl Palmitate	0.79 ± 0.10 **AB**b	0.97 ± 0.09 **A**bcd	0.73 ± 0.04 **B**defg	0.81 ± 0.09 **AB**a	0.87 ± 0.02 **AB**cde	0.95 ± 0.08 **AB**abc	1.02 ± 0.06 **A**b	0.81 ± 0.08 **AB**c	0.84 ± 0.07 **AB**b
Methyl Oleate	0.83 ± 0.11 **BC**b	1.10 ± 0.12 **AB**bcd	0.69 ± 0.13 **C**efg	0.76 ± 0.05 **C**a	0.90 ± 0.02 **ABC**bcde	0.89 ± 0.09 **ABC**abc	1.17 ± 0.13 **A**b	0.71 ± 0.05 **C**c	0.96 ± 0.06 **ABC**b
Methyl Linoleate	0.65 ± 0.10 **C**b	0.83 ± 0.06 **BC**d	0.75 ± 0.11 **BC**defg	0.97 ± 0.03 **BC**a	0.91 ± 0.02 **B**bcde	0.81 ± 0.03 **BC**abc	1.17 ± 0.14 **A**b	0.80 ± 0.05 **BC**c	0.80 ± 0.05 **BC**b
Methyl Linolenate	0.62 ± 0.12 **C**b	0.98 ± 0.08 **AC**bcd	0.62 ± 0.11 **C**d	0.78 ± 0.04 **BC**a	0.87 ± 0.01 **ABC**cde	0.81 ± 0.03 **BC**abc	1.11 ± 0.16 **A**b	0.71 ± 0.08 **BC**c	0.82 ± 0.04 **BC**b
Methyl Stearate	0.70 ± 0.18 **B**b	0.94 ± 0.04 **AB**bcd	0.76 ± 0.12 **AB**defg	0.77 ± 0.09 **AB**a	0.84 ± 0.05 **AB**e	0.75 ± 0.05 **AB**bc	1.07 ± 0.14 **A**b	0.77 ± 0.04 **AB**c	0.85 ± 0.05 **AB**b
Ethyl Palmitate	0.67 ± 0.13 **C**b	0.78 ± 0.06 **BC**d	0.67 ± 0.09 **C**fg	0.80 ± 0.06 **BC**a	0.89 ± 0.03 **ABC**bcde	0.76 ± 0.04 **BC**bc	1.10 ± 0.16 **A**b	0.78 ± 0.03 **BC**c	0.95 ± 0.06 **AB**b
Ethyl Oleate	0.67 ± 0.09 **C**b	0.78 ± 0.10 **BC**d	0.67 ± 0.09 **C**fg	0.80 ± 0.04 **BC**a	0.89 ± 0.04 **ABC**bcde	0.77 ± 0.05 **BC**bc	1.10 ± 0.07 **A**b	0.78 ± 0.03 **BC**c	0.95 ± 0.16 **AB**b
Ethyl Linoleate	0.68 ± 0.10 **B**b	1.33 ± 0.23 **A**b	1.10 ± 0.22 **AB**bcde	0.79 ± 0.04 **B**a	0.93 ± 0.02 **AB**bcde	0.78 ± 0.05 **B**bc	1.03 ± 0.13 **AB**b	0.88 ± 0.05 **B**bc	0.87 ± 0.14 **B**b
Ethyl Stearate	0.67 ± 0.13 **B**b	1.07 ± 0.08 **A**bcd	1.06 ± 0.12 **A**bcdef	0.81 ± 0.04 **A**Ba	0.99 ± 0.06 **AB**abc	0.82 ± 0.05 **AB**abc	0.97 ± 0.18 **AB**b	0.90 ± 0.05 **AB**bc	0.83 ± 0.12 **AB**b
Linalool	0.77 ± 0.18 **A**b	0.77 ± 0.14 **A**d	0.90 ± 0.15 **A**cdefg	0.82 ± 0.05 **A**a	0.99 ± 0.02 **A**abcd	0.85 ± 0.04 **A**abc	1.11 ± 0.13 **A**b	1.01 ± 0.06 **A**bc	1.01 ± 0.13 **A**b
Ocimene	0.63 ± 0.23 **BC**b	1.29 ± 0.13 **BC**bc	1.14 ± 0.13 **BC**bcd	0.77 ± 0.10 **BC**a	0.31 ± 0.10 **C**f	0.70 ± 0.22 **BC**c	3.83 ± 0.92 **A**a	1.63 ± 0.41 **B**a	1.75 ± 0.20 **B**a
Ethyl Hexanoate	0.72 ± 0.14 **B**b	0.95 ± 0.10 **B**bcd	1.33 ± 0.15 **A**b	0.85 ± 0.08 **B**a	1.03 ± 0.02 **AB**ab	0.89 ± 0.05 **B**abc	1.05 ± 0.13 **AB**b	0.94 ± 0.07 **B**bc	0.83 ± 0.16 **B**b
Farnesene (mixture of isomers)	0.78 ± 0.04 **B**b	1.27 ± 0.11 **A**bc	1.24 ± 0.16 **A**bc	0.95 ± 0.09 **A**Ba	1.09 ± 0.02 **AB**a	0.95 ± 0.05 **AB**abc	1.13 ± 0.15 **A**b	1.03 ± 0.05 **AB**bc	1.00 ± 0.10 **AB**b
Nonanal	1.56 ± 0.39 **ABC**a	1.86 ± 0.23 **AB**a	1.96 ± 0.15 **A**a	0.97 ± 0.03 **C**a	1.09 ± 0.04 **BC**a	1.02 ± 0.06 **C**ab	1.62 ± 0.38 **ABC**b	1.27 ± 0.14 **ABC**b	1.70 ± 0.34 **ABC**a

The data in the table are relative ELG response ratios ± SE. The lowercase letters indicate the significance between different volatiles under the same organization (same column), and the bold uppercase letters indicate the significance between different organizations (peers) of the same volatile (*p* < 0.05, LSD).

## Data Availability

The data supporting the findings of this study are available from the corresponding author upon reasonable request.
